# Advantages of Synthetic Noise and Machine Learning for Analyzing Radioecological Data Sets

**DOI:** 10.1371/journal.pone.0170007

**Published:** 2017-01-09

**Authors:** Igor Shuryak

**Affiliations:** Center for Radiological Research, Columbia University, New York, New York, United States of America; Shanxi University, CHINA

## Abstract

The ecological effects of accidental or malicious radioactive contamination are insufficiently understood because of the hazards and difficulties associated with conducting studies in radioactively-polluted areas. Data sets from severely contaminated locations can therefore be small. Moreover, many potentially important factors, such as soil concentrations of toxic chemicals, pH, and temperature, can be correlated with radiation levels and with each other. In such situations, commonly-used statistical techniques like generalized linear models (GLMs) may not be able to provide useful information about how radiation and/or these other variables affect the outcome (e.g. abundance of the studied organisms). Ensemble machine learning methods such as random forests offer powerful alternatives. We propose that analysis of small radioecological data sets by GLMs and/or machine learning can be made more informative by using the following techniques: (1) adding synthetic noise variables to provide benchmarks for distinguishing the performances of valuable predictors from irrelevant ones; (2) adding noise directly to the predictors and/or to the outcome to test the robustness of analysis results against random data fluctuations; (3) adding artificial effects to selected predictors to test the sensitivity of the analysis methods in detecting predictor effects; (4) running a selected machine learning method multiple times (with different random-number seeds) to test the robustness of the detected “signal”; (5) using several machine learning methods to test the “signal’s” sensitivity to differences in analysis techniques. Here, we applied these approaches to simulated data, and to two published examples of small radioecological data sets: (I) counts of fungal taxa in samples of soil contaminated by the Chernobyl nuclear power plan accident (Ukraine), and (II) bacterial abundance in soil samples under a ruptured nuclear waste storage tank (USA). We show that the proposed techniques were advantageous compared with the methodology used in the original publications where the data sets were presented. Specifically, our approach identified a negative effect of radioactive contamination in data set I, and suggested that in data set II stable chromium could have been a stronger limiting factor for bacterial abundance than the radionuclides ^137^Cs and ^99^Tc. This new information, which was extracted from these data sets using the proposed techniques, can potentially enhance the design of radioactive waste bioremediation.

## Introduction

The ecological consequences of radioactive contamination after an accident at a nuclear power plant or nuclear waste storage site remain poorly understood because it can be difficult and hazardous to conduct studies in contaminated areas. Due to these limitations, data sets collected from locations where the contamination is most severe are often small and study design can be sub-optimal. However, even small data sets collected under field conditions can in principle provide useful information which would be difficult to obtain in the laboratory.

Such complexity of ecological data can make their analysis challenging. For example, the abundance of studied organisms in soil can be affected, not only by the severity and composition of contamination, but also by other environmental variables. Identifying the effects of these factors can provide insight into predicting and understanding the ecological impact of radioactive contamination, and potentially into mitigating its impact by bioremediation.

Generalized linear models (GLMs) are a popular tool for analyzing ecological data. GLM-based analysis can be enhanced by using multi-model inference (MMI), which involves using potentially relevant predictor variables to construct multiple models and quantify the strength of support from the data for each model based on information theory (e.g. using the sample-size-corrected Akaike information criterion, AICc). The models can then be combined by model averaging, where each model’s contribution is weighted by its support. Compared with using a single model, this procedure generally produces more robust parameter estimates and uncertainties. Another very useful property of MMI is that predictor variables, which were present within the evaluated set of models, can be ranked by their importance. For example, a predictor which is consistently present in all highly-supported models can be considered much more important than another predictor which appears only in poorly-supported models.

However, in ecological data sets some (or even most) of the potential predictors can be irrelevant for describing the outcome. Some predictors can have effects which are too small to be reliably detected at the available sample size. MMI often assigns relatively small, but nevertheless non-zero importance to such variables. Consequently, there is no easily identifiable “threshold” for distinguishing valuable predictors from noise variables.

An intuitive solution for this problem is to introduce into the data set some artificially-generated noise variables [1] which “mimic” the real predictors. Analyzing the data set with such synthetic noise variables being included allows the researcher to identify those predictors which achieve higher importance than the noise variables. In other words, artificial noise variables serve as “benchmarks” for distinguishing “signal” from “noise”.

Another useful way to utilize synthetic noise is to apply it directly to the predictors. For example, continuous predictor variables can be modified by adding to them some normally-distributed random values. This approach enables the researcher to estimate the robustness of analysis conclusions in the presence of random fluctuations in the data: more robust conclusions will, up to a point, withstand larger fluctuations. In addition, artificial effects of selected predictors on the outcome can be introduced. This artificially strengthens the association between the selected predictors and the outcome. Using this method, the researcher can estimate how large does the effect of a selected predictor need to be in order to become detectable by the analysis procedure, given the limitations (e.g. small sample size) of the data set at hand.

The approaches using GLMs and MMI can work reasonably when the number of predictors is much smaller than the number of observations. However, often the number of potentially relevant predictor variables can be similar to or even exceed the number of observations. In such situations, GLMs either cannot be fitted at all or can provide very inaccurate parameter estimates.

Ensemble methods in machine learning offer powerful alternatives. Briefly, they train and test multiple models of a given type (e.g. decision trees or GLMs) on randomly-selected subsets of the data, and combine the results. In this manner they generate more robust and accurate predictions than would be possible using a single model [2]. They can handle situations where the number of predictors is larger than the number of observations, and/or most of these predictors are in fact irrelevant.

The following three machine learning methods (summarized in Table 1) may represent good options for handling those radioecological data sets, which prove difficult to analyze by GLMs:

1. Random generalized linear models (RGLMs) [3] uses GLMs as base models. Predictor variables for the GLMs are selected by minimizing the Akaike information criterion (AIC). Bootstrap aggregation (bagging) is used to extend this approach as an ensemble method. This relatively recent machine learning algorithm was reported to have state-of- the-art performance[3].
2. Random forests (RF) [4] have become very popular for both classification and regression problems. This method uses decision trees as base models, and employs bagging and tree de-correlation approaches to improve performance. Decision trees have some very useful properties for analyzing noisy data sets with different predictor types and large ratio of predictors to observations: they are robust against outliers and to the presence of many irrelevant predictors, and are unaffected by monotonic (e.g. logarithmic) transformations of the predictors [2].
3. Generalized boosted regression modeling (abbreviated as GB here for convenience) [2] also use decision trees. Unlike RF, however, trees are averaged by boosting rather than bagging. Boosting involves iterative fitting of trees: the data are reweighted so that the next trees focus more strongly on those data points on which previous trees performed poorly.

**Table 1:** Summary of ensemble machine learning methods employed here.

Method	Base models	Ensemble-building approach	References
Random generalized linear models (RGLM)	Generalized linear models	Bagging	[3]
Random forests (RF)	Decision trees	Bagging	[4]
Generalized boosted regression models (GB)	Decision trees	Boosting	[2, 5, 6]

A commonly-occurring problem in ecological data sets is collinearity [7]: predictors can be strongly correlated with each other. For example, the concentrations of several types of radionuclides and toxins, which were released from a common source, can be correlated in soil samples. GLM-based analysis of data sets with strong collinearity can produce unreliable parameter estimates and uncertainties and therefore can lead to misleading interpretations about the effect of any given predictor. Tree-based methods, such as RF or GB, are more robust in the presence of collinearity [7]. However, to mitigate the effects of collinearity even further it can be useful to remove those predictors which: (1) are strongly correlated to other predictors (e.g. Pearson correlation coefficient > 0.7) and/or (2) have the highest variance inflation factors (VIF) [7].

Here, we use the methods described above (GLMs, MMI, machine learning, and synthetic noise) on two published examples of small radioecological data sets. These examples are interesting because they provide information about the effects of very severe radioactive contamination on soil microorganisms under field conditions. As mentioned previously, data sets of this type are quite rare due to the hazards of working in heavily contaminated locations.

The first data set consists of numbers of fungal taxa isolated from several locations near the Chernobyl nuclear power plant during the first 5 years after the accident [8]. This is a unique data set because it describes how fungi were affected by Chernobyl fallout shortly after the event. The second data set consists of the abundance of bacteria in soil contaminated by a leaked radioactive waste storage tank at the Hanford site, near Richland, Washington [9]. To our knowledge, this is the only detailed published study of the effects of high-level nuclear waste on soil bacteria. Both data sets are described in more detail in the Materials and Methods section, and supplied in S1 and S2 Appendices, respectively.

Our analysis of these data sets using the proposed methodology led to new conclusions. These conclusions may have useful implications for understanding and predicting the effects of radioactive contamination on soil microorganisms, and for developing bioremediation strategies.

## Materials and Methods

### Synthetic noise variables as benchmarks

Suppose a continuous predictor variable X(*i*) for the *i*-th observation (where the number of observations is *i* = 1, 2..N) has a mean μ and a standard deviation σ in the analyzed data set. A synthetic noise variable X_n_(*i*) can be generated by drawing random numbers from the normal distribution with parameters μ and σ. Noise variables can be produced by an analogous procedure for other types of predictors, for example by using other distributions (e.g. Bernoulli instead of normal for binary variables) or by bootstrapping with replacement (for categorical ones).

These synthetic noise variables can be added as extra predictors to the data set and analyzed by GLMs and MMI along with real predictors. The importances of real and synthetic variables can then be compared to identify which (if any) real predictors perform better than synthetic ones. In other words, the synthetic variables help to establish a “benchmark” for separating those predictors with strong explanatory power from those whose effects cannot be reliably distinguished from noise.

This procedure can be implemented as follows, where VIM(X) is the variable importance measure calculated for predictor X by the selected analysis procedure, VIM_min_ is the minimum VIM value achieved by the lowest-scoring predictor, VIM_NoiseMax_ is the VIM value achieved by the highest-scoring synthetic noise variable, and VIMr(X) is the VIM of predictor X relative to synthetic noise variables:

$$\text{VIMr}\left(\text{X}\right)\;=\;\left(\text{VIM}\left(\text{X}\right)\;-{\text{ VIM}}_{\text{min}}\right)/\left({\text{VIM}}_{\text{NoiseMax}}-{\text{ VIM}}_{\text{min}}\right)$$ (1)

The metric VIMr(X) is convenient because it represents an easily interpretable ratio for the performance of predictor X relative to synthetic noise. For example, VIM(X) in RF can be measured by %IncMSE, which is the percentage increase in mean squared error of predictions when the predictor X is permuted. Therefore, VIM(X) and/or VIM_min_ calculated by RF can be negative, but VIMr(X) will be positive and will have the following intuitive interpretation: If VIMr(X) > 1, predictor X has higher importance than any of the synthetic noise variables.

### Synthetic noise added to predictor and outcome variables

Synthetic noise can be introduced into the data not only in the form of extra variables, but can also be applied directly to the real predictors and/or to the outcome. For example, a continuous predictor variable X(*i*) can be modified as follows, where ν is the magnitude of random noise, SN is a random number drawn from the standard normal distribution (with mean = 0 and standard deviation = 1), and X_m_(*i*) is the predictor with added noise:

$${\text{X}}_{\text{m}}\left(i\right)\;=\text{ X}\left(i\right)\;+\;\nu \times \text{SN}$$ (2)

Analyzing the data set with noise included in this manner allows the researcher to test the sensitivity of analysis results to random perturbations of the predictors. The magnitude of the noise (ν) can be increased to see how much noise can be tolerated by the procedure used for analyzing the data before its performance degrades. For example, suppose that when the unperturbed data set was analyzed, a certain set of predictors had importances above those of synthetic noise variables. Adding increasing amounts of noise to the system estimates the level of noise at which the importances of these predictors begin to overlap those of noise variables. The critical noise level at which a given predictor’s effect can no longer be detected can differ for different predictors—those that can “tolerate” higher noise levels may (although not necessarily) contain a stronger “signal”.

### Artificially strengthening the effects of selected predictors

It is also potentially useful to assess the ability of the analysis procedure to detect “signals” in the data set at hand by artificially strengthening the effects of certain predictors. For example, suppose the outcome is a continuous variable Y(*i*). It can be modified as follows, where η is the effect magnitude of predictor X(*i*):

$${\text{Y}}_{\text{m}}\left(i\right)\;=\text{ Y}\left(i\right)\;+\eta \times \text{X}\left(i\right)$$ (3)

In an unperturbed situation where η = 0, X(*i*) may have a low importance (within the range of noise variables). By increasing η the researcher can test how large the effect of X(*i*) would need to be in order to be detected within the analyzed data set (i.e. at the available sample size and in the presence of other predictors) by the selected analysis method. The impact of noise can also be tested in the same manner by replacing X(*i*) with X_m_(*i*). In other words, the researcher can vary ν and η to evaluate the robustness of analysis results with respect to detecting signals in the data.

### Randomizing the outcome

Randomizing the outcome variable (e.g. by permuting or bootstrapping it with replacement) provides the opposite test by creating a situation where no signals are present. The analysis procedure should detect only noise under such conditions. In other words, the importances of all predictors should be in the same range as those of noise variables.

### Running each machine learning algorithm multiple times

The results of ensemble machine learning methods applied to the same data set can depend on the random number seed. Consequently, it was shown that repeating the machine learning analysis several times with different random number seeds is more reliable than a single run [10, 11]. Specifically, running a machine learning algorithm multiple times with different seeds generates a distribution of VIMr values across runs. For example, suppose that based on 1000 runs, predictor X_1_ has a mean VIMr of 2.0 (range: 1.2, 3.5), whereas predictor X_2_ has a mean VIMr of 0.5 (range: 0.1, 1.1). This result suggests that X_1_ consistently (on average) outperforms noise variables, whereas X_2_ generally performs no better than noise, but can occasionally achieve high importance scores by chance.

For convenience, we will use the following notation for variable importance scores: Mean VIMr across multiple machine learning runs is abbreviated as mVIMr. Values of mVIMr > 1, which suggest that the variable on average outperformed noise, are called “high mVIMr”. In contrast, values of mVIMr < 1, which suggest that the variable on average did not outperform noise, are called “low mVIMr”.

The robustness of mVIMr estimates of course is expected to increase with increasing number of random runs. For small data sets 1000 runs are computationally feasible within reasonable time, and therefore we used this number of runs throughout our calculations.

### Techniques used to avoid overfitting

Overfitting of the data can be an important problem both for parametric models such as GLMs, and for machine learning algorithms. It occurs when not only the underlying relationships in the analyzed data set, but also the random errors, are fitted. The result can be very good performance of the analysis method on the selected data set, but poor performance on other data sets: i.e. poor generalization from training data to test data.

For tree-based machine learning methods such as RF, overfitting generally occurs if the trees are allowed to continue splitting to purity [10, 11]. In other words, if the trees are allowed to become very complex, they are likely to “overreact” to noise in the data. A simple solution for this problem involves preventing the terminal nodes in any tree from containing less than about 10% of the sample size [10, 11]. This critical value (Nc) can be calculated simply by rounding the product 0.1×N, where N is the sample size. Of course, this cutoff approach is only a rough approximation—a monotonically increasing function of sample size may yield better results, but at the cost of having additional tunable parameters. For convenience, here we applied the simple restriction on Nc to all three machine learning methods: RGLM, RF and GB.

### Combining machine learning with parametric models

Machine learning methods are sometimes considered to be “black boxes” which are good at making predictions, but do not summarize the patterns which they extracted from the data in an easily-interpretable form. Recent developments, which assist the visualization and interpretation of machine learning output (e.g. the *forestFloor* R package), seek to dispel this view. However, we believe that it can also be useful to combine machine learning with traditional parametric models (e.g. linear regression). For example, machine learning can be used to identify the best predictors in the data (e.g. those with high mVIMr), and these predictors can then be used to construct an easily-interpretable parametric model. This two-step procedure has the following advantages in situations where the number of predictors is comparable to (or larger than) the number of observations: (1) Machine learning detects predictors with strong “signals”, allowing weak and irrelevant ones to be discarded. This step can be repeated to ensure that a consistent set of predictors is retained. (2) The remaining predictor set becomes small enough to be handled by a parametric model, which then generates interpretable parameter estimates.

### Software used for data analysis

To analyze the selected data sets, we used R software (version 3.2.3). A sample R code and additional details are provided in S3 Appendix. As mentioned previously, we focused the data analyses on the search for predictors with strong main effects, rather than on interactions. However, to assess whether or not any very important pairwise interactions were missed we used the *find*.*interaction* algorithm in the *randomForestSRC* package [12–14].

### Simulated data sets

Before analyzing the real radioecological data sets which were introduced above and will be described in more detail below, we performed exploratory calculations to examine the performances of the selected machine learning methods on three different simulated data sets. These data sets, as well as the results obtained when they were analyzed, are described in S4 Appendix.

### Radioecological data sets

The first data set consists of numbers of fungal taxa isolated from soil near the Chernobyl nuclear power plant during the first 5 years after the accident [8]. Briefly, soil samples at depths of 0–10 cm were collected close to the Chernobyl nuclear power plant over the period of 1987 to 1991 [8]. Fungi were isolated from the samples by culturing on Czapek's or wort agar with added antibiotics. There were six sampling locations (coded by letters A-F), which included Chernobyl town (E), two villages (B, C), two forests (A, D), and the outskirts of a large city (F). Radioactive contamination (in Bq/kg) was measured in the top layer of soil (0–2 cm) and in a deeper layer (8–10 cm) at each location. The dominant radionuclides were ^137^Cs, ^134^Cs, ^144^Ce, ^141^Ce, ^106^Ru, ^103^Ru. Other potentially relevant environmental variables reported in the study were: soil pH, depth, and year of sampling [8].

For our analysis, we extracted the numbers of fungal taxa (TotalTaxa) at each location from Fig 4 of reference [8]. Depth in soil (Depth) was coded as 1 for 0–2 cm and as 9 for 8–10 cm. Time of sampling (Year) was coded as 87 for 1987–88 and as 89 for 1989–91. The severity of radioactive contamination and soil pH were extracted from Table 2 of reference [8]. The radioactivity was reported as a range of activities (in Bq/kg), e.g. 1.6×10^4^–7.8×10^6^ for the 0–2 cm soil layer at location A [8]. We summarized this information by calculating the average of log-transformed activities (coded as AvLogRad). For this specific example, AvLogRad = [log(1.6×10^4^)+log(7.8×10^6^)]/2 = 5.55.

These data certainly represent only a small part of the information collected by the authors of reference [8]. However, they were useful for our purposes here—we sought to test the ability of our proposed approach of using synthetic noise to identify valuable predictors specifically on small and limited-quality radioecological data sets.

Consequently, the data set we analyzed (S1 Appendix) contained TotalTaxa as the outcome, and 5 predictors: Location, Depth, AvLogRad, pH, and Year. As described previously, for each of these predictors we created 5 synthetic noise variables. Synthetic “analogs” of continuous predictors AvLogRad and pH were generated by drawing random numbers from the normal distribution with the same mean and standard deviation as calculated for the measured variable. Depth and Year were binary (codes as 1 or 9, or 87 or 89, respectively). Consequently, synthetic “analogs” of these variables were drawn from the Bernoulli distribution with probability of 0.5: results of 0 were assigned to the smallest code (e.g. 1 for depth and 87 for year) and results of 1 were assigned to the largest code (9 for depth and 89 for year). Location was a categorical variable (letters A-F), and therefore synthetic “analogs” were created by drawing uniformly-distributed random numbers between 0 and 1 and assigning results between 0 and 1/6 to letter A, 1/6 to 2/6 to letter B, etc.

The second data set was generated by bacteriological analysis of vadose sediments located under a high-level radioactive waste storage tank at the Hanford site, near Richland, Washington [9]. Briefly, sixteen soil samples were collected at various depths beneath the tank 40–50 years after it leaked, releasing radionuclides and chemical contaminants [9]. Only one contaminated site was sampled, but we believe that it is sufficiently representative to support a modeling analysis. Two control samples were obtained from a nearby unpolluted location. Temperature, pH, water content, conductivity, concentrations of ^137^Cs, ^99^Tc, Cr, NO_3_ and NO_2_ were recorded for each sample [9]. Aerobic heterotrophic bacteria were isolated from the soil samples on peptone-tryptone-yeast extract-glucose agar (PTYG), and their abundance was represented by log colony forming units (CFU) per gram.

For our analysis of this data set, we extracted the number of viable bacteria (log[CFU/g]) isolated by culturing on PTYG agar from Table 3 and from the main text of reference [9].These outcome data were coded as variable logCFU. For samples where no bacteria were detected (listed as BD in reference [9]), we assigned logCFU = -2 (but also performed sensitivity calculations with logCFU = 1). The potential predictors were extracted from Table 2 and from the main text of reference [9]. They were: vertical depth (m) in soil (depth), percent water content (water), pH (pH), conductivity in mS/cm (log-transformed to logCond), ^137^Cs in nCi/g (log-transformed to logCs), Cr in μg/g (log-transformed to logCr), NO_3_ in mg/L (log-transformed to logNO3) and NO_2_ in mg/L (log-transformed to logNO2). Data for ^99^Tc in pCi/L were extracted from Fig 2 of reference [9] and log-transformed to logTc. The log transformations were performed on variables which varied by several orders of magnitude among the samples. For NO_2_, Table 2 of reference [9] contains some entries of “< 1”, “< 10” or BD. We coded these values as 0.5, 5, and 0.05, respectively and then log-transformed them. For un-contaminated control samples, we assigned values of -4 to logCs, logTc, logCr, logNO3 and logNO2.

**Table 2:** Poisson regression analysis of data set I (fungi at Chernobyl). VIF represents gVIF^x^, where x = 1/(2×dof) and dof is the number of degrees of freedom. For the categorical variable Location which had 6 levels (A-F), VIF was the same (1.6) for all levels. Details about the data set and calculations are provided in the main text.

Variable	Poisson regression				MMI			
	Coefficient	SE	p-value	VIF	Coefficient	SE	p-value	Importance
Intercept	-12.700	9.693	0.190		-7.292	45.4		
LocationB	-0.085	0.407	0.834	1.6	-0.002	0.151	0.988	0.003
LocationC	-0.327	0.684	0.633		0.003	0.199	0.990	0.003
LocationD	-19.399	1683.5	0.991		-0.012	0.288	0.967	0.003
LocationE	-1.287	1.638	0.432		0.005	0.336	0.989	0.003
LocationF	-1.677	1.782	0.347		0.004	0.342	0.991	0.003
Depth	-0.021	0.036	0.556	1.3	-0.011	0.104	0.917	0.204
AvLogRad	-0.888	0.323	0.006	2.6	-1.340	1.105	0.226	0.725
pH	0.229	0.426	0.591	5.7	0.020	0.306	0.949	0.197
Year	0.198	0.109	0.070	1.0	0.188	0.510	0.712	0.266

**Table 3:** Linear regression analysis of data set II (bacteria at Hanford). Details about the data set and calculations are provided in the main text.

Variable	Linear regression				MMI			
	Coefficient	SE	p-value	VIF	Coefficient	SE	p-value	Importance
Intercept	8.386	16.550	0.626		1.197	3.009	0.691	
Depth	-0.146	0.255	0.584	8.01	0.004	0.030	0.893	0.08
Water	0.033	0.176	0.855	2.44	0.007	0.039	0.856	0.09
pH	-0.726	1.427	0.625	3.46	-0.057	0.302	0.849	0.09
logCond	-1.032	2.602	0.702	8.46	-0.006	0.223	0.980	0.06
logCs	-0.744	0.831	0.397	11.42	-0.021	0.125	0.864	0.10
logTc	0.512	1.881	0.792	102.11	0.023	0.175	0.896	0.11
logCr	-0.224	1.346	0.872	20.20	-0.641	0.498	0.198	0.74
logNO3	0.413	2.685	0.881	112.36	0.026	0.219	0.907	0.12
logNO2	-1.793	1.542	0.278	17.19	-0.269	0.549	0.625	0.31

Consequently, the second data set we analyzed (S2 Appendix) contained logCFU as the outcome, and 9 predictors: depth, water, pH, logCond, logCs, logTc, logCr, logNO3 and logNO2. For each of these predictors, 5 synthetic noise variables were generated by drawing random numbers from the normal distribution with the same mean and standard deviation as calculated for the measured variable.

## Results

### Data set I: Fungi at Chernobyl

**Fig 1:**
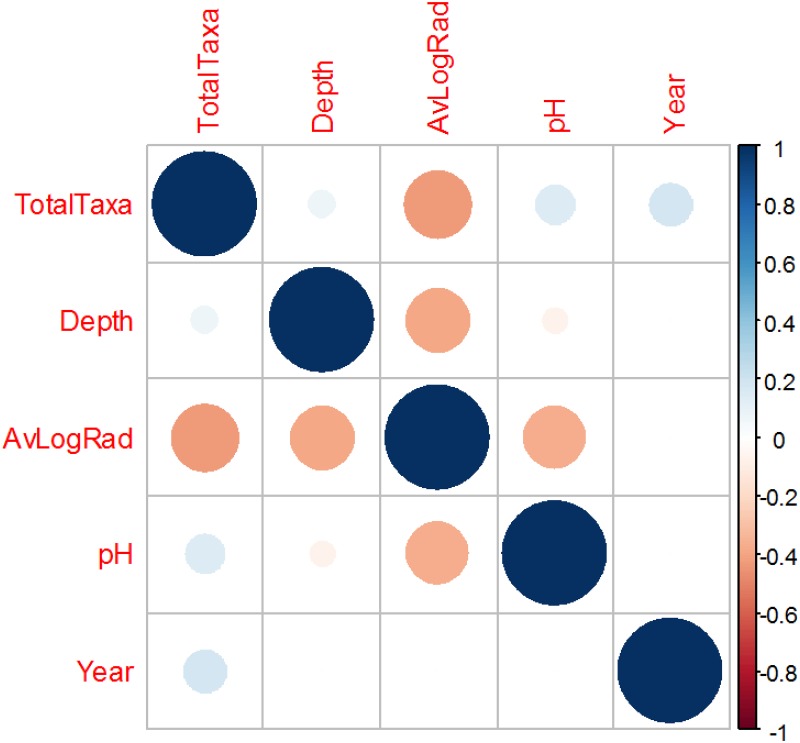
Graphical representation of Pearson correlations between numerical variables in data set I (fungi at Chernobyl).

The number of fungal taxa isolated from soil at various locations near the Chernobyl nuclear power plant during the first five years after the accident (the outcome, called TotalTaxa) had a moderate negative correlation with the severity of radioactive contamination (AvLogRad) (Fig 1). The other numerical predictor variables (Depth, pH and Year) had small positive correlations with the outcome (Fig 1). The Mantel test revealed no auto-correlation of the number of taxa with depth in soil (p-value = 0.98).

Poisson regression showed that AvLogRad had a statistically significant negative effect (p-value = 0.006), whereas all other predictors did not achieve statistical significance (Table 2). MMI assigned the highest importance to AvLogRad, followed by Year and Depth (Table 2). Because there was evidence for collinearity (high VIF values, Table 2), we removed from the model the variable pH which had the highest VIF. Poisson regression using the remaining four predictors produced the following coefficients: Depth = -0.012 (SE: 0.032, p-value = 0.69), AvLogRad = -0.752 (SE: 0.192, p-value = 9.1×10^−5^), Year = 0.198 (SE: 0.109, p-value = 0.07). The coefficients for each location (A-F) were non-significant (p-values ≥ 0.11). There was no longer any evidence of substantial collinearity (VIF values corrected for degrees of freedom were 1.0–1.5). The goodness of fit of this simplified model was reasonable: pseudo R^2^ values estimated by Cragg and Uhler's and maximum likelihood methods were 0.91 and 0.92, respectively. MMI again assigned the highest importance (0.74) to AvLogRad, followed by Year (0.27).

However, as mentioned previously, such results do not provide enough information for separating valuable predictors from those which may have achieved a non-negligible importance merely by chance. Adding synthetic noise variables to the data set provides benchmarks which indicate what magnitudes of importance can be obtained by chance. In this specific data set, we added two random variables per each real variable, as described above. For example, the random variables R1AvLogRad and R2AvLogRad were random numbers drawn from the normal distribution with the same mean and standard deviation as the real variable AvLogRad. Therefore, the data set with benchmarks contained 12 predictors: Location, Depth, AvLogRad, Year, and two random “mimics” of each of these variables.

In this data set, which included synthetic random variables, the three most important real variables were AvLogRad (importance = 0.75), Year (0.16) and Depth (0.13). The three most important random variables were: R2Year (0.59), R1AvLogRad (0.19) and R2AvLogRad (0.18). These results suggest that the importance of only one real variable (AvLogRad) was well above the values achieved by noise variables. The parameter estimate for AvLogRad was -1.35.

Location B (Chistogolovka village), which was one of the two most contaminated sites, had the sparsest counts of fungal taxa: no taxa were detected in the top 0–2 cm of soil over the entire study period, and even in the deeper layer of 8–10 cm some taxa were found only in 1989–91, but not in 1987–88 [8]. To assess what effect the data at this site had on variable importances, we performed the analysis on the data set with location B excluded. Still, the most important variable (with a value of 0.59) was AvLogRad, whereas the highest-scoring noise variable (R2Year) had a value of 0.29. The parameter estimate for AvLogRad was -1.09. Therefore, removing the data for location B did not qualitatively alter the conclusion about the negative effect of radioactive contamination on fungal taxa counts.

Partitioning of the data into darkly-pigmented (melanized) and lightly-pigmented taxa and separate analyses of each of these groups led to the same conclusions, although the errors of course increased (e.g. for the less numerous lightly-pigmented taxa even the importance of AvLogRad fell within the range for noise variables).

In summary, analysis of this data set using GLMs (Poisson regression) suggested that the most important predictors of fungal taxa numbers were the level of radioactive contamination (AvLogRad) and time after the Chernobyl accident (Year). However, collinearity of predictors may have distorted these results. The proposed methodology (reduction of collinearity by removal of the variable with the highest VIF, and introduction of synthetic noise variables as benchmarks for MMI-estimated predictor importance) indicated that only the first of these variables (AvLogRad) consistently outperformed noise variables. In other words, the proposed methodology provided more confidence in the conclusion that the level of radioactive contamination was the most influential predictor in this data set.

### Data set II: Bacteria at Hanford

**Fig 2:**
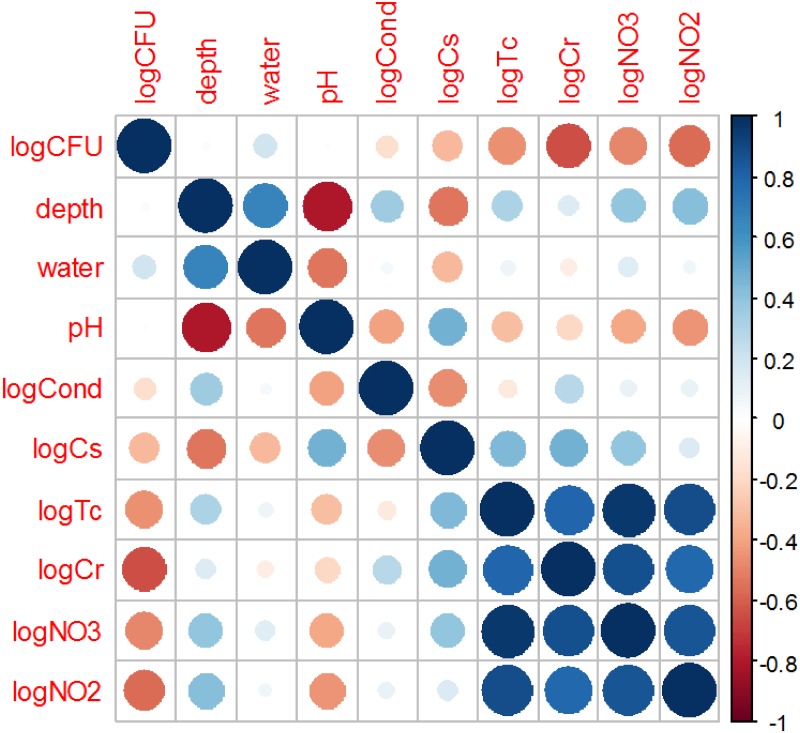
Graphical representation of Pearson correlations between numerical variables in data set II (bacteria at Hanford).

The Mantel test revealed no auto-correlation of bacterial abundance (logCFU) with depth in soil (depth): p-value = 0.50.Water content (water) had a slight positive correlation with logCFU, conductivity (logCond) had a slight negative one, whereas pH had essentially no correlation (Fig 2). Negative correlations with logCFU were found for all measured contaminants: ^137^Cs (logCs), ^99^Tc (logTc), Cr (logCr), NO_3_ (logNO3) and NO_2_ (logNO2) (Fig 2). Interestingly, the strongest negative correlation was detected for Cr, rather than for the two radionuclides (^137^Cs and ^99^Tc).

It is not surprising that many contaminants (especially logTc, logCr, logNO3 and logNO2, Fig 2) were positively correlated with each other, because they were all released from a common source—the leaked nuclear waste tank. This situation can lead to the problem of collinearity, where the results of data analysis can be distorted [7]. Linear regression performed on this data set indeed suggested the presence of collinearity: several predictors had very high VIF values (Table 3). None of the predictors reached statistical significance.

MMI assigned the highest importance (0.74) to logCr, and the next-ranking predictor was logNO2 (0.31). Because these results are unlikely to be reliable in the presence of collinearity, we performed the linear regression and MMI analyses again, after removing two predictors with the highest VIFs: logTC and logNO3. However, many of the remaining predictors (depth, logCond, logCs, logCr and logNO2) still had VIFs > 5. The importances from MMI remained similar to the ones obtained on the full data set: 0.70 for logCr and 0.37 for logNO2.

These results suggested that the performance of linear regression on this data set was degraded by collinearity. As mentioned previously, machine learning methods can be more robust in the presence of collinearity [7]. Here, we used the three methods described above: RGLM, RF and GB. When the unperturbed original data set was analyzed, all three methods consistently assigned the highest importance to logCr. For example, in RF the importance of a given predictor was measured by %IncMSE, as mentioned previously. Across 1000 RF runs with different initial random seeds, logCr had a mean importance score of 13.99 (SD: 0.76, range: 11.27, 15.97). The next ranking predictor was logCs with a score of 9.36 (SD: 0.82, range: 6.67, 11.83).

Some predictors (e.g. depth, water, logCond) had negative mean importance scores from RF, e.g. -3.19 (SD: 0.93, range: -5.83, -0.03) for water. The addition of synthetic random variables to the data set, as described above, provided benchmarks to identify the range of importance scores which is likely to be achieved by noise variables. Each predictor’s performance could then be compared to the performance of noise variables. This approach showed than only logCr consistently outperformed the noise variables: its mean relative importance score (VIMr) from RF was 1.39 (SD: 0.14, range: 1.02, 1.90). In other words, logCr had (on average) a 1.39-fold higher importance than the top-ranking noise variable.

The results were qualitatively similar for RGLM and GB: logCr outperformed noise by factors of 1.06 (SD: 0.05, range: 0.89, 1.24) and 1.75 (SD: 0.29, 1.07, 3.14), respectively. In contrast, the VIMr scores of the next ranking predictor, logCs, were 0.93 (SD: 0.11, range: 0.54) for RF, 0.15 (SD: 0.02, range: 0.09, 0.21) for RGLM, and 0.69 (SD: 0.17, range: 0.30, 1.56) for GB. All other predictors had even worse performance, with mean scores ≤ 0.56 for all three machine learning methods.

**Fig 3:**
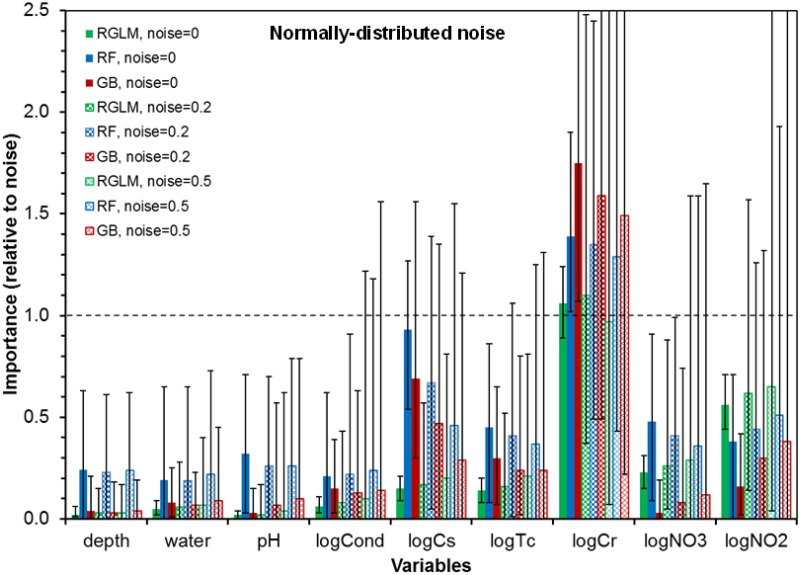
Summary of machine learning analyses of data set II (bacteria at Hanford) by the RGLM, RF and GB methods. The x-axis lists predictor variables, and the y-axis displays VIMr (bars = mean values for 1000 runs with different random number seeds, error bars = range). VIMr values > 1 indicate that the particular predictor achieved higher importance than any of the synthetic noise variables. The analyses were repeated under different amounts of normally-distributed noise (ν), as listed in the legend. Details are described in the main text.

**Fig 4:**
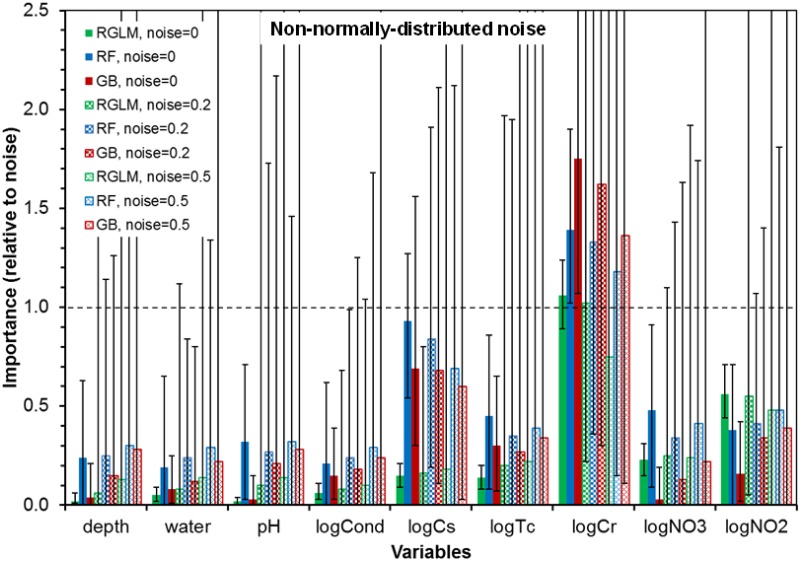
Summary of machine learning analyses of data set II (bacteria at Hanford) by the RGLM, RF and GB methods conducted under different amounts of non-normally-distributed noise (ν), as listed in the legend. The meanings of the axes and variables are the same as in Fig 3. Details are described in the main text.

Adding various amounts of noise (either normally or non-normally distributed) to the predictors and to the outcome expectedly broadened the ranges of scores attained by each predictor, but did not change the overall pattern: only logCr robustly outperformed noise variables (Figs 3 and 4). For all three machine learning methods, logCr maintained mVIMr > 1 when the noise added to all predictors and the outcome had a magnitude of ν = 0.2. When the noise magnitude was increased to 0.5, mVIMr for logCr dropped to 0.97 for RGLM, but remained >1 for RF and GB (Fig 3). Using the GB method, mVIMr of logCr was exceptionally robust: to reduce it to < 1, we had to: (*a*) increase ν to 1.0 for all variables, or (*b*) set ν to 2.5 for logCr while keeping it at 0.5 for all other variables. Qualitatively similar results were obtained for non-normally (log-normally) distributed noise (Fig 4).

The robustness of the “signal” from logCr was maintained when the outcome variable (logCFU) was systematically modified. As explained previously, in some soil samples no viable bacteria were detected, and we assigned logCFU = -2 for these instances. If instead we assigned logCFU = 1, the results of analyzing the data by the three machine learning methods (without or with noise) remained qualitatively the same: only logCr achieved higher importance than noise variables.

An alternative test, where logCFU was randomly bootstrapped with replacement, was performed to “remove the signal” from the data set. In this situation, all three machine learning methods produced similar mVIMr scores for all real and synthetic noise variables. For example, the mVIMr for logCr from GB was only 0.18 (SD: 0.31, range: 0.00, 5.12), and the scores for all other predictors were also ≤ 0.2. The patterns were similar for RF and RGLM. Adding noise did not qualitatively alter these results. In other words, when all predictors were rendered meaningless by randomizing the outcome, all three machine learning methods detected only noise, as expected. No spurious effects of any of the predictors were found.

**Fig 5:**
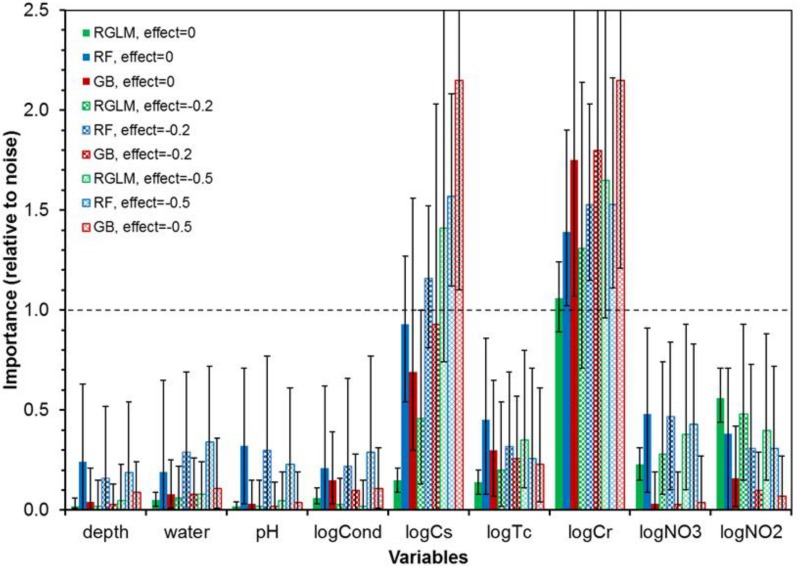
The effects of artificially strengthening the effects of predictor logCs (the log-transformed concentration of ^137^Cs) on machine learning analyses of data set II (bacteria at Hanford). The magnitudes of artificial effects (η) are listed in the legend. The meanings of the axes and variables are the same as in Figs 3 and 4. Details are described in the main text.

These analyses using various forms of noise all pointed to logCr as the most informative predictor of logCFU. Somewhat unexpectedly, the dominant radionuclide ^137^Cs consistently achieved lower importance scores, within the range of noise variables. To estimate how large would the effect of ^137^Cs need to be in order to be detected in this data set by the selected machine learning methods, we added artificial effects of logCs on logCFU. The results showed that an effect coefficient of η = -0.2 was too small to result in high mVIMr for logCs, whereas a coefficient of -0.5 was sufficiently large (Fig 5). Therefore, the artificial effect did not need to be very large (e.g. not much larger than the coefficient estimated by linear regression, Table 3) to boost the importance of logCs above the range of scores from noise variables. This suggests that the lower scores attained by logCs when no artificial effects were introduced are not likely caused by low sensitivity of the machine learning methods, but are likely due to weak “signal” from this variable. In contrast, logCr had a stronger “signal” which stood out above the noise.

Similar results were found when artificial effects were added for logNO3 or for both logCs and logNO3. Modestly-sized negative effect coefficients were needed to increase mVIMr values of one or both of these variables to > 1. Throughout these analyses, logCr maintained high mVIMr, i.e. its performance was not substantially affected by the performances of logCs or logNO3.

We expected that the ratio (ξ) of synthetic random variables to measured predictor variables would affect mVIMr scores as follows: High ξ values would produce conservative results, where only those predictors with very strong effects would achieve high mVIMr, whereas those with weaker effects could be obscured by noise. Low ξ values, on the other hand, could allow detection of weak effects, but the confidence in separating signal from noise would be decreased (i.e. irrelevant variables could be mistakenly assigned high mVIMr).

Exploratory calculations on data set II where both ξ and the noise level ν were varied were consistent with these expectations. For example, at ν = 0.2, the GB method showed that only logCr achieved mVIMr > 1 when ξ was 2, 3 or 5. However, when ξ was reduced to 1, logCs also achieved mVIMr > 1. The same pattern occurred at ν = 0. The RF method assigned high mVIMr to logCs, logTc, logCr, and logNO3 at ξ = 1, but only to logCr at ξ≥2. This occurred at either ν = 0 or 0.2. The RGLM method assigned high mVIMr to logCr, and logNO2 at ξ = 1, but only to logCr at ξ≥2. This also occurred at either ν = 0 or 0.2. Therefore, at no-to-moderate noise level (ν = 0 or 0.2), two synthetic variables per measured variable were sufficient to identify logCr as the only predictor with high mVIMr according to all three machine learning methods. In contrast, using one synthetic variable per measured variable allowed other predictors to achieve high mVIMr as well. Because the list of these predictors varied among machine learning methods (e.g. logNO3 was selected by RF, whereas logNO2 was selected by RGLM), it is likely that their high mVIMr values are spurious. By comparison, the consistently high mVIMr values of logCr across multiple ξ levels and machine learning methods suggest that this variable may indeed have a strong association with bacterial abundance.

To further assess the potential impact of collinearity on the machine learning results, we repeated the analysis on data sets from which those predictors, which were strongly correlated to other predictors, were removed. For example, when logTc was removed, the only remaining predictor which had mVIMr > 1 was logCr: this occurred at ν = 0 and at ν = 0.2. The same pattern (i.e. mVIMr > 1 for logCr) was repeated when logNO2 or logNO3 were removed, or when logCs, logNO3 and logNO2 were removed together. However, when logCr was removed, none of the remaining predictors achieved high mVIMr. These other predictors achieved mVIRr > 1 only if artificial effects were added, which suggested that their effects in the unperturbed data set were too weak to be reliably detected.

A search for pairwise interactions between predictors using RF, as described above, showed that the importance score achieved by any pair of predictors was essentially identical to the sum of individual scores from these predictors. In other words, no important pairwise interactions were detected. This result appears mechanistically plausible, because the predictors represent radionuclides, chemical toxins and abiotic environmental factors, and it appears likely that for each of these variables the main effect should be more important than interactions.

Our analysis of this data set therefore suggested that the predictor with the strongest “signal”, which could be separated from noise by the utilized machine learning methods, was logCr. Linear regression where logCFU was the outcome and logCr was the only predictor produced the following parameters: intercept = 1.18 (SE: 0.56, p-value = 0.05), logCr coefficient = -0.82 (SE: 0.25, p-value = 0.004), R^2^ = 0.41. Therefore, logCr explained a non-negligible amount of the variance in the data.

In summary, analysis of this data set using GLMs (linear regression) produced inconclusive results, probably due to collinearity of several predictors (Table 3). In contrast, the proposed methodology (the use of machine learning methods coupled with the introduction of several forms of synthetic noise) indicated that only one predictor, logCr, consistently outperformed noise variables. In other words, whereas standard GLM analysis did not provide much useful information, the proposed methodology identified a putative influential predictor for bacterial abundance in the polluted soil: the concentration of Cr. Results of this type can be used to focus future research on the effects of nuclear waste on soil microbes.

## Discussion

Here we proposed that distinguishing predictors with strong main effects from less important/irrelevant ones in radioecological data sets can be made easier by the inclusion of synthetic noise variables. These synthetic variables provide benchmarks for how noise is expected to perform in the analyzed data set. Only those predictors which consistently outperform the noise are likely to contain valuable “signals”.

Our preliminary calculations using simulated data (S4 Appendix) showed that this approach, performed reasonably well. For example, even if the data set was small (e.g. 20 observations) and noisy, and the number of potential predictors exceeded the number of observations, predictors with strong main effects (high mVIMr) could be identified. In contrast, predictors with weak or non-existent effects correctly achieved low mVIMr. These results were quite robust in the presence of noise applied to the predictor variables and/or to the outcome.

Our analysis of a real data set (data set I) on fungal taxon counts in various locations near the Chernobyl nuclear power plant accident site [8] detected a strong negative effect of radioactive contamination (AvLogRad). There was also some evidence that the number of fungal taxa increased as more time passed after the accident, but this effect was not strong enough to be detected with confidence.

These results were not specifically reported in reference [8], which was concerned mainly with the species composition of fungal communities at sampled locations. However, they are mechanistically plausible: higher radiation dose rates are likely to decrease the number of taxa by eliminating the most radiosensitive ones; whereas longer time after the accident decreases the dose rate and allows colonization of contaminated sites by fungi dispersing through the air from other locations. Consequently, we believe that our analysis methods worked adequately in this case, even though the data set was small (only 24 observations). Importantly, these methods resulted in new findings (such as the negative effect of radioactive contamination on fungal taxon numbers), which were not reported in reference [8].

The second real data set (data set II) on bacterial abundance in soil near a ruptured nuclear waste tank [9] was even smaller (18 observations), and contained many correlated predictors: radionuclides and chemical toxins released from the tank. Due to these limitations, and to the generally low bacterial abundance in the studied soil, the authors of reference [9] found it “difficult to discern trends in either population size or presence of aerobic heterotrophic bacteria in relation to sediment properties such as pH, water content, and contaminant concentration”.

In other words, data set II was not statistically analyzed in reference [9]. Here we performed a detailed statistical analysis of this data set using linear regression, MMI and machine learning algorithms. Linear regression (even strengthened by MMI) did not produce informative results: collinearity of predictors most likely strongly distorted parameter estimates and this collinearity could not be mitigated by dropping predictors based on VIF values. In contrast, machine learning algorithms suggested that some “signal” can be extracted from this data set. Specifically, all three methods (RGLM, RF and GB) identified logCr as the only predictor which on average outperformed synthetic noise variables. This result remained unchanged when the most correlated predictors were dropped and/or when noise was added to the predictors and to the outcome. When logCr was dropped, no other predictor was able to achieve high mVIMr. This result, together with the failure to detect important interactions by RF, suggested that the high importance scores of logCr are not likely to be due to variable interactions, but may in fact represent a strong main effect of Cr. In other words, Cr concentration was selected as the strongest predictor of bacterial abundance. When used as the only predictor in a linear model, logCr had a strong negative effect on logCFU and explained a non-negligible portion of the data variability: R^2^ = 0.41.

Therefore, the analysis methods proposed and implemented here resulted in a new finding—the effect of Cr—which was not reported in reference [9]. Although Cr can be highly toxic to bacteria [15], we did not expect that it would be the only predictor with high importance because the other measured constituents of nuclear waste also have strong toxicity. Nevertheless, in this data set other potentially relevant predictors, such as the dominant radionuclide ^137^Cs (logCs), did not consistently outperform noise variables. If the effect of logCs was artificially strengthened by a moderate amount, it could be detected (i.e. logCs achieved high mVIMr). This suggested that failure to detect a strong effect of logCs in unperturbed data is unlikely to be due to insufficient sensitivity of the machine learning methods, but is more likely due to weakness of the effect.

Therefore, the stable metal Cr (and/or some unreported pollutants which may have been correlated with it) may have been a stronger limiting factor for bacterial abundance in this data set than the radionuclides ^137^Cs and ^99^Tc, or nitrogen compounds. This is a potentially important conclusion which can enhance the understanding of nuclear waste effects on soil bacteria and assist the design of bioremediation efforts.

In summary, the proposed approach allows useful information to be extracted even from small and limited-quality radioecological data sets. Specifically, this approach was advantageous compared with the methodology used by the authors of references [8, 9] because it identified important patterns (i.e. the effect of radioactive contamination in data set I and the effect of Cr in data set II) which were not reported in the original publications.

However, some important limitations of the proposed methodology need to be mentioned. First, the information obtained from statistical techniques such as those used here is of course only correlational. Potential causal relationships need to be investigated by more focused experimental studies and/or by mechanistic modeling. Second, there are no clear-cut guidelines for selecting the level of synthetic noise introduced into the data. For example, should one choose a 1:1 ratio of synthetic noise variables to measured predictors, or higher ratios such as 2:1, 5:1, etc? Very high ratios may produce overly “conservative” results, such that it becomes increasingly difficult to detect the effect of any predictor. Here, using data set II as an example, we found evidence that a ≥2:1 ratio of synthetic noise variables to measured predictors may give more reliable results, than a 1:1 ratio. Third, although synthetic noise is useful as a benchmark and for assessing the robustness of analysis results, such noise may either trigger a fake effect or may hinder a true effect. Therefore, the conclusions drawn from analyzing radioecological data sets using the methods outlined here should preferably be validated by further studies.

Therefore, the proposed methodology is by no means definitive, but it can help to distinguish "signal" from "noise", thereby providing a clue about potentially important predictors that may need further verification. Specifically, such methodology can focus the researcher's attention on those variable(s) which are associated with the outcome strongly enough to outperform noise. These variables can then be evaluated further by additional studies.

## Supporting Information

S1 Appendix Data set I (fungi at Chernobyl). (XLSX)

S2 Appendix Data set II (bacteria at Hanford). (XLSX)

S3 Appendix R software and code used for machine learning analyses. (DOCX)

S4 Appendix Simulated data sets. (DOCX)
