# Supplementary material for: Advantages of Synthetic Noise and Machine Learning for Analyzing Radioecological Data Sets
Source: PLoS One. 2017 Jan 9;12(1):e0170007. doi: 10.1371/journal.pone.0170007 (PMC5222373; doi:10.1371/journal.pone.0170007)
Supplement: S3 Appendix — (DOCX) [file pone.0170007.s003.docx]

**R software and code used for machine learning analyses**

As mentioned in the main text, to analyze the selected data sets by the RGLM, RF and GB machine learning methods, we used R software (version 3.2.3). For RGLM, we used the following options: *classify* = FALSE (i.e. running in regression mode rather than in classification mode), *maxInteractionOrder* = 1 (searching for main effects), *nBags* = 100 (using 100 random subsets of the data), *minInBagObs* = Nc. The metric used as VIM was *timesSelectedByForwardRegression*, i.e. the number of times when a given predictor was included in the model based on AIC. For RF, we used: *ntree* = 1000 (building 1000 trees), *importance* = TRUE (using *%IncMSE* as the VIM measure), *nodesize* = Nc. For GB, we used: *distribution* = "gaussian" (the Gaussian error distribution is assumed), *n.trees* = 1000 (building 1000 trees), *cv.folds* =10 (performing 10 fold cross validation), interaction.depth = 1 (searching for main effects), *n.minobsinnode* = Nc, *shrinkage* = 0.005 (also known as learning rate, 0.001 to 0.1 are recommended). The metric used as VIM was relative influence.

A sample R program code to implement the RGLM, RF and GB machine learning methods on data set II is provided below.

# needed R packages

require(randomGLM)

require(randomForest)

require(gbm)

require(biganalytics)

require(corrplot)

# reading data files

vars = read.csv(file.choose( )) # file containing matrix of predictor variables (each variable in a separate column, with rows corresponding to observations)

logCFU = read.csv(file.choose( )) # file containing vector of observed outcome (logCFU) values

# transforming the input files from data frame format to matrix format for convenience

varsm = as.matrix(vars)

logCFUm = as.matrix(logCFU)

# numbers of variables in data set

obs = 18 # number of observations

numvars = 54 # total number of variables: real (measured) predictors plus synthetic noise

numrealvars = 9 # total number of real (measured) predictors

# input parameters for synthetic noise

nu = 0 # synthetic noise level

nuspecial = 1 # extra noise multiplier for special selected variable(s)

eta = 0 # special effect coefficient for selected variable(s)

# calculating and plotting Pearson correlations for real variables

varsreal = data.frame(logCFU, vars[ -c(numrealvars+1:numvars) ])

varsrealcor <- cor(varsreal)

corrplot(varsrealcor, method = "circle")

# number of random machine learning simulations (default = 1000)

numsims = 1000

# creating matrices to store output from machine learning methods

RFres1 = matrix(0, numsims, numrealvars)

RGLMres1 = matrix(0, numsims, numrealvars)

GBres1 = matrix(0, numsims, numrealvars)

# performing simulations

for (i in 1:numsims) {

# setting random number seed

set.seed=111*i

# adding normally distributed noise to all predictor variables: real (measured) and synthetic noise (those starting with R1..R5)

depthnoise = with(vars, depth+nu*rnorm(obs))

waternoise = with(vars, water+nu*rnorm(obs))

pHnoise = with(vars, pH+nu*rnorm(obs))

logCondnoise = with(vars, logCond+nu*rnorm(obs))

logCsnoise = with(vars, logCs+nu*rnorm(obs))

logTcnoise = with(vars, logTc+nu*rnorm(obs))

logCrnoise = with(vars, logCr+nuspecial*nu*rnorm(obs))

logNO3noise = with(vars, logNO3+nu*rnorm(obs))

logNO2noise = with(vars, logNO2+nu*rnorm(obs))

R1depthnoise = with(vars, R1depth+nu*rnorm(obs))

R2depthnoise = with(vars, R2depth+nu*rnorm(obs))

R3depthnoise = with(vars, R3depth+nu*rnorm(obs))

R4depthnoise = with(vars, R4depth+nu*rnorm(obs))

R5depthnoise = with(vars, R5depth+nu*rnorm(obs))

R1waternoise = with(vars, R1water+nu*rnorm(obs))

R2waternoise = with(vars, R2water+nu*rnorm(obs))

R3waternoise = with(vars, R3water+nu*rnorm(obs))

R4waternoise = with(vars, R4water+nu*rnorm(obs))

R5waternoise = with(vars, R5water+nu*rnorm(obs))

R1pHnoise = with(vars, R1pH+nu*rnorm(obs))

R2pHnoise = with(vars, R2pH+nu*rnorm(obs))

R3pHnoise = with(vars, R3pH+nu*rnorm(obs))

R4pHnoise = with(vars, R4pH+nu*rnorm(obs))

R5pHnoise = with(vars, R5pH+nu*rnorm(obs))

R1logCondnoise = with(vars, R1logCond+nu*rnorm(obs))

R2logCondnoise = with(vars, R2logCond+nu*rnorm(obs))

R3logCondnoise = with(vars, R3logCond+nu*rnorm(obs))

R4logCondnoise = with(vars, R4logCond+nu*rnorm(obs))

R5logCondnoise = with(vars, R5logCond+nu*rnorm(obs))

R1logCsnoise = with(vars, R1logCs+nu*rnorm(obs))

R2logCsnoise = with(vars, R2logCs+nu*rnorm(obs))

R3logCsnoise = with(vars, R3logCs+nu*rnorm(obs))

R4logCsnoise = with(vars, R4logCs+nu*rnorm(obs))

R5logCsnoise = with(vars, R5logCs+nu*rnorm(obs))

R1logTcnoise = with(vars, R1logTc+nu*rnorm(obs))

R2logTcnoise = with(vars, R2logTc+nu*rnorm(obs))

R3logTcnoise = with(vars, R3logTc+nu*rnorm(obs))

R4logTcnoise = with(vars, R4logTc+nu*rnorm(obs))

R5logTcnoise = with(vars, R5logTc+nu*rnorm(obs))

R1logCrnoise = with(vars, R1logCr+nu*rnorm(obs))

R2logCrnoise = with(vars, R2logCr+nu*rnorm(obs))

R3logCrnoise = with(vars, R3logCr+nu*rnorm(obs))

R4logCrnoise = with(vars, R4logCr+nu*rnorm(obs))

R5logCrnoise = with(vars, R5logCr+nu*rnorm(obs))

R1logNO3noise = with(vars, R1logNO3+nu*rnorm(obs))

R2logNO3noise = with(vars, R2logNO3+nu*rnorm(obs))

R3logNO3noise = with(vars, R3logNO3+nu*rnorm(obs))

R4logNO3noise = with(vars, R4logNO3+nu*rnorm(obs))

R5logNO3noise = with(vars, R5logNO3+nu*rnorm(obs))

R1logNO2noise = with(vars, R1logNO2+nu*rnorm(obs))

R2logNO2noise = with(vars, R2logNO2+nu*rnorm(obs))

R3logNO2noise = with(vars, R3logNO2+nu*rnorm(obs))

R4logNO2noise = with(vars, R4logNO2+nu*rnorm(obs))

R5logNO2noise = with(vars, R5logNO2+nu*rnorm(obs))

# combining all predictors with noise into data frame

varsnoise = data.frame(depthnoise, waternoise, pHnoise, logCondnoise, logCsnoise, logTcnoise, logCrnoise, logNO3noise, logNO2noise, R1depthnoise, R1waternoise, R1pHnoise, R1logCondnoise, R1logCsnoise, R1logTcnoise, R1logCrnoise, R1logNO3noise, R1logNO2noise, R2depthnoise, R2waternoise, R2pHnoise, R2logCondnoise, R2logCsnoise, R2logTcnoise, R2logCrnoise, R2logNO3noise, R2logNO2noise, R3depthnoise, R3waternoise, R3pHnoise, R3logCondnoise, R3logCsnoise, R3logTcnoise, R3logCrnoise, R3logNO3noise, R3logNO2noise, R4depthnoise, R4waternoise, R4pHnoise, R4logCondnoise, R4logCsnoise, R4logTcnoise, R4logCrnoise, R4logNO3noise, R4logNO2noise, R5depthnoise, R5waternoise, R5pHnoise, R5logCondnoise, R5logCsnoise, R5logTcnoise, R5logCrnoise, R5logNO3noise, R5logNO2noise )

# adding normally distributed noise to outcome variable

logCFUnoise1 = with(logCFU, logCFU+nu*rnorm(obs))

logCFUnoise = data.frame( logCFUnoise1 )

# compiling data for convenience

X = varsnoise

Y = logCFUnoise

XYtot = data.frame(Y,X)

# adding a special effect for variable logCs

Ymod = with(XYtot, logCFUnoise + eta*logCsnoise)

# compiling data for convenience

Xm = as.matrix(X)

Ym = as.matrix(Ymod)

# performing RF analysis and calculating importance scores

rf1 = randomForest (

Xm, #columns of predictor variables

Ym, #column of outcome variable

keep.inbag = TRUE,

importance = TRUE, # calculating importance scores

nodesize = round(0.1*obs, digits = 0), # using cutoff of 10% of sample size to avoid overfitting

ntree = 1000, setseed=111*i)

rf1imp = data.frame(round(importance(rf1), 3))

rf1imp$X.IncMSE[1:numrealvars] # importances of real variables

minimptot = min(rf1imp$X.IncMSE[1:numvars], na.rm=TRUE) # min importance for any variable

noisemax = max(rf1imp$X.IncMSE[numrealvars+1:numvars], na.rm=TRUE)-minimptot # max importance for noise variables

# performing RGLM analysis and calculating importance scores

RGLMv1 = randomGLM(Xm, Ym, maxInteractionOrder=1, classify=FALSE, replace=TRUE, minInBagObs=round(0.1*obs, digits = 0), nCandidateCovariates=round(numrealvars/1.5, digits = 0), keepModels=TRUE, nBags=100, randomSeed=111*i)

RGLMvarImpv1 = data.frame(RGLMv1$timesSelectedByForwardRegression)

RGLMnoisemax = max(RGLMvarImpv1[(numrealvars+1):numvars], na.rm=TRUE)

# performing GB analysis and calculating importance scores

XYtot2 = data.frame(Ym,Xm)

gbmfit1 = gbm(logCFUnoise1 ~ ., data=XYtot2, distribution = "gaussian", n.trees = 1000, cv.folds=10, interaction.depth = 1, n.minobsinnode = round(0.1*obs, digits = 0), shrinkage = 0.005, bag.fraction = 0.5, keep.data = FALSE)

GBimpmat = as.matrix(relative.influence(gbmfit1, n.trees=1000))

GBminimptot = min(GBimpmat[1:numvars], na.rm=TRUE)

GBnoisemax = max(GBimpmat[numrealvars+1:numvars], na.rm=TRUE)-GBminimptot

# compiling variable importance scores from RGLM, RF and GB methods

for (j in 1:numrealvars) {

RFres1[i,j] = (rf1imp$X.IncMSE[j]-minimptot)/noisemax

RGLMres1[i,j] = max(RGLMvarImpv1[j])/RGLMnoisemax

GBres1[i,j] = (abs(GBimpmat[j])-GBminimptot)/GBnoisemax

}

}

# output of results (VIMr values) from RGLM, RF and GB methods

# the order of the 9 measured predictor variables is: depth, water, pH, logCond, logCs, logTc, logCr, logNO3, logNO2

RFres1b = as.big.matrix(RFres1)

colmean(RFres1b, na.rm=TRUE) # RF VIMr values, mean

colsd(RFres1b, na.rm=TRUE) # RF VIMr values, sd

colmin(RFres1b, na.rm=TRUE) # RF VIMr values, min

colmax(RFres1b, na.rm=TRUE) # RF VIMr values, max

RGLMres1b = as.big.matrix(RGLMres1)

colmean(RGLMres1b, na.rm=TRUE) # RGLM VIMr values, mean

colsd(RGLMres1b, na.rm=TRUE) # RGLM VIMr values, sd

colmin(RGLMres1b, na.rm=TRUE) # RGLM VIMr values, min

colmax(RGLMres1b, na.rm=TRUE) # RGLM VIMr values, max

GBres1b3 = as.big.matrix(GBres1)

colmean(GBres1b3, na.rm=TRUE) # gradient boosting VIMr values, mean

colsd(GBres1b3, na.rm=TRUE) # gradient boosting VIMr values, sd

colmin(GBres1b3, na.rm=TRUE) # gradient boosting VIMr values, min

colmax(GBres1b3, na.rm=TRUE) # gradient boosting VIMr values, max
