# Supplementary material for: Advantages of Synthetic Noise and Machine Learning for Analyzing Radioecological Data Sets
Source: PLoS One. 2017 Jan 9;12(1):e0170007. doi: 10.1371/journal.pone.0170007 (PMC5222373; doi:10.1371/journal.pone.0170007)
Supplement: S4 Appendix — (DOCX) [file pone.0170007.s004.docx]

**Simulated data sets**

**Construction of data sets**

We performed exploratory calculations to examine the performances of the selected machine learning methods on the following simulated data sets:

1. A data set with only one real predictor and many noise variables. This scenario was used to test how well the selected methods distinguish the real predictor from multiple noise variables under various sample sizes and noise levels. There were *j* = 1,2…90 predictor variables X*_j_*(*i*), where *i* is the observation index. The response variable Y(*i*) was generated as follows, where X_5_(*i*) was the only real predictor, η was the magnitude of its effect, ν was the magnitude of noise, and SN was a random number drawn from the standard normal distribution:

Y(*i*) = η×X_5_(*i*) + ν×SN (1)

All predictors, except X_5_(*i*), were random numbers (SN). The noise term ν×SN was applied to all 90 predictors and to the outcome variable. The first 15 predictors X*_1_*(*i*),X*_2_*(*i*)...X*_15_*(*i*) were intended to represent the number of variables which could be measured in a radioecological study (e.g. concentrations of various radionuclides, environmental variables). The remaining 75 predictors were intended to represent synthetic random variables which mimic these measured variables to provide benchmarks for variable importance, as described above.

1. A data set with 10 real predictors with tapering effect sizes. The effect sizes of the first 10 predictors were η = 1, -1, ½, -½, ¼, -¼, 1/8, -1/8, 1/16 and -1/16, respectively. Predictors from 11 to 90 had zero effect. The other properties of the data set were similar to those of the first one. This scenario was used to test what effect sizes can be detected by the selected machine learning methods, when tapering effects of multiple predictors are present, as can be the case in real data sets [1].
2. A data set with correlated and non-normally distributed predictors. The first predictor was a standard normal random variable X_1_(*i*) = SN, whereas the second one X_2_(*i*) was binary (0 or 1), drawn from a Bernoulli distribution with a probability of ½. The outcome variable was generated from these predictors as follows, where UN is a random number drawn from the uniform distribution between 0 and 1:

Y(*i*) = X_1_(*i*)+X_2_(*i*)+ ½×UN (2)

Consequently, the only real predictors were 1 and 2.

However, to produce a more complicated situation for testing the machine learning algorithms, we created additional non-normally distributed predictors which had no effect on the outcome, but could be correlated with real predictors. Specifically, we defined:

X_3_(*i*) = X_1_(*i*)×exp[½×(UN – ½)] (3)

X_4_(*i*) = [(X_2_(*i*) + ¼)×(1 + UN)]^½^ (4)

X_5_(*i*) = exp[X_3_(*i*) + UN – ½] (5)

X_6_(*i*) = X_4_(*i*)/(1 + UN) (6)

X_7_(*i*) = (1+ UN)^2^ (7)

The predictors 1, 3 and 5 had strong positive correlations with the outcome and/or with each other. For each of the 7 predictors defined above, 5 synthetic random variables were constructed, bringing the total number of predictors to 7+5×7 = 42. This scenario was used to test the effects of deviations from the normal distribution and predictor correlations on the performances of machine learning methods.

**Analysis results**

The selected machine learning methods performed well at identifying the real predictor among lots of noise in the first simulated data set. For example, even if the data set was small (N=20 observations) and fairly noisy (ν=0.2), an effect size of η=0.2 for the real predictor X_5_(*i*) was detectable: mVIMr from 1000 runs of GB was 2.57 (SD: 2.14, range: 0.02, 15.38). A smaller effect size of η=0.1 was on the verge of being detected: mVIMr = 0.83 (SD: 0.97, range: 0.00, 7.53).

When many predictors had tapering effect sizes (in the second simulated data set), only the strongest ones (i.e. predictors 1 and 2, with effects of 1 and -1, respectively) could be detected in small and noisy data (N=20, ν=0.2). In other words, only predictors 1 and 2 had high mVIMr for RGLM, RF and GB (where high mVIMr refers to values >1, as described above). The results for these strongest predictors were robust: very strong noise (ν=1.0) was needed to reduce their mVIMr values to <1. Of course, at no noise (ν=0) and larger sample size (N=200) all machine learning methods detected even weaker predictors: RF assigned high mVIMr to predictors 1-4, and RGLM and GB assigned high mVIMr to predictors 1-6.

When many predictors were not normally distributed and could be correlated (simulated data set three), and the data set was small and noisy (N=20, ν=0.2), RGLM and GB assigned high mVIMr to predictors 1, 2, 3, and 5. RF differed by assigning a slightly lower score of 0.60 (SD: 0.21, range: 0.00, 1.65) to predictor 2. In other words, the machine learning methods identified one (but not always both) real predictors (1 and 2). However, they also assigned high importance to some predictors (3 and 5) which were correlated with the outcome, but in fact had no effect. These results were very robust with regard to noise, and tolerated even ν=1.0.

**References**

1. Burnham KP, Anderson DR. Model selection and multi-model inference: a practical information-theoretic approach: Springer; 2002.
